# Supplementary material for: Anti-Inflammatory Dietary Diversity and Depressive Symptoms among Older Adults: A Nationwide Cross-Sectional Analysis
Source: Nutrients. 2022 Nov 28;14(23):5062. doi: 10.3390/nu14235062 (PMC9736199; doi:10.3390/nu14235062)
Supplement: Supplementary file 1 [file nutrients-14-05062-s001.zip › nutrients-1971590-supplementary.pdf]

Supplement Table S1：Sensitivity Analyses of associations of types of Dietary Diversity, Dietary Pattern with depressive symptoms.

	Diet Diversity Index (DDI)	Protein-enriched Diet Diversity Index (PEDDI)	Anti-inflammatory Diet Diversity Index (AIDDI)
**MMSE⩾21**			
Model 1	0.88 (0.87-0.90) *	0.87 (0.85-0.90) *	0.76 (0.74-0.79) *
Model 2	0.90 (0.88-0.92) *	0.90 (0.88-0.93) *	0.78 (0.76-0.81) *
Model 3	0.91 (0.89-0.93) *	0.91 (0.88-0.94) *	0.80 (0.77-0.83) *
Model 4	0.91 (0.89-0.92) *	0.91 (0.88-0.93) *	0.80 (0.77-0.83) *
**Without ADL**			
Model 1	0.88 (0.86-0.90) *	0.87 (0.85-0.90) *	0.75 (0.73-0.78) *
Model 2	0.89 (0.88-0.91) *	0.90 (0.87-0.93) *	0.77 (0.75-0.80) *
Model 3	0.91 (0.89-0.92) *	0.91 (0.88-0.94) *	0.79 (0.77-0.82) *
Model 4	0.90 (0.88-0.92) *	0.90 (0.87-0.93) *	0.79 (0.76-0.82) *
**No Imputation**			
Model 1	0.88 (0.87-0.90) *	0.88 (0.85-0.90) *	0.77 (0.74-0.79) *
Model 2	0.90 (0.88-0.92) *	0.90 (0.88-0.93) *	0.78 (0.76-0.81) *
Model 3	0.91 (0.89-0.93) *	0.91 (0.89-0.94) *	0.80 (0.78-0.83) *
Model 4	0.91 (0.89-0.93) *	0.91 (0.88-0.94) *	0.80 (0.77-0.83) *

Model 1 controlling for gender, age;

Model 2 controlling for gender, age, hukou, ethnic, BMI, education;

Model 3 controlling for gender, age, hukou, ethnic, BMI, education, physical activities, smoking, drinking;

Model 4 controlling for gender, age, hukou, ethnic, BMI, education, smoking, drinking, feeding in ADL disability, physical activities, dementia, tooth count.

* $P<0.05$.
